# A role for *EHMT2* in a novel autosomal recessive neurodevelopmental syndrome? A case report

**DOI:** 10.3389/fgene.2026.1824138

**Published:** 2026-06-26

**Authors:** Dmitrijs Rots, Beatriz Cristina de Oliveira, Laura Machado Lara Carvalho, Xiaonan Zhao, Bekim Sadikovic, Teresa Sim, Robert Rigobello, Matthew Tedder, Sarah Donoghue, Devi Priyanka Maripuri, Ales Hnizda, Eileen Barr, Robin Fletcher, Lenka Noskova, Dong Li, Tjitske Kleefstra, Elaine H. Zackai, Maria J. Barrero, Ana Cristina Victorino Krepischi, Alanna Strong

**Affiliations:** 1 Department of Clinical Genetics, Erasmus MC, Rotterdam, Netherlands; 2 Department of Genetics and Evolutionary Biology, Institute of Biosciences, Human Genome and Stem Cell Research Center, University of Sao Paulo (USP), Sao Paulo, Brazil; 3 Cajal Institute, Spanish National Research Council (CSIC), Madrid, Spain; 4 Baylor Genetics, Houston, TX, United States; 5 Department of Molecular and Human Genetics, Baylor College of Medicine, Houston, TX, United States; 6 Verspeeten Clinical Genome Center, London Health Sciences Center, London, ON, Canada; 7 Department of Pathology and Laboratory Medicine, Western University, London, ON, Canada; 8 Greenwood Genetic Center, Greenwood, SC, United States; 9 Division of Human Genetics, Children’s Hospital of Philadelphia, Philadelphia, PA, United States; 10 Center for Applied Genomics, Children’s Hospital of Philadelphia, Philadelphia, PA, United States; 11 Research Unit for Rare Diseases, Department of Pediatrics and Inherited Metabolic Disorders, 1st Faculty of Medicine and General University Hospital, Charles University in Prague, Prague, Czechia; 12 Department of Pediatrics, Perelman School of Medicine at the University of Pennsylvania, Philadelphia, PA, United States; 13 Department of Human Genetics, Radboud University Medical Center, Nijmegen, Netherlands; 14 Center of Excellence for Neuropsychiatry, Vincent van Gogh Institute for Psychiatry, Venray, Netherlands; 15 Institute of Rare Diseases Research (IIER), Spanish National Institute of Health Carlos III (ISCIII), Madrid, Spain; 16 Undiagnosed Diseases Program (SpainUDP), Madrid, Spain

**Keywords:** candidate gene, case report, *EHMT2*, Kleefstra syndrome, multi-omics

## Abstract

**Background:**

*EHMT1* and *EHMT2* encode histone methyltransferases that form an epigenetic complex mediating mono- and dimethylation of histone H3 at lysine 9 (H3K9me1/2). This complex modulates fundamental biological processes during embryonic and post-natal development. While *EHMT1* has an established role in neurodevelopmental disease, with heterozygous pathogenic variants causing Kleefstra syndrome type 1 (KS1), the contribution of *EHMT2* to neurodevelopmental disorders remains to be established. To date, seven probands harboring *de novo* heterozygous *EHMT2* variants and one individual with a homozygous splice variant have been reported, all presenting with phenotypes and DNA methylation episignatures overlapping with KS1.

**Methods:**

A male proband was referred for Genetics evaluation due to global developmental delay, autism spectrum disorder, hypotonia, dysmorphisms, posterior fossa malformation, congenital heart disease, umbilical hernia, and genitourinary anomalies. Trio genome sequencing identified compound heterozygous variants in *EHMT2* (NM_006709.5:c.2648_2649del; p.(Glu883Glyfs*48), paternally inherited; NM_006709.5:c.2344-19_2344-16del; r.spl, maternally inherited). DNA methylation episignature profiling and RNA-sequencing were performed to assess the molecular consequences of these *EHMT2* variants.

**Results:**

Proband phenotype strongly overlapped with that of KS1 and previously reported individuals with autosomal dominant and recessive *EHMT2*-related neurodevelopmental disorder. DNA methylation episignature was consistent with KS1. Transcripts bearing the paternally inherited *EHMT2* frameshift variant were under-represented in the RNA-sequencing data, likely reflecting partial nonsense-mediated decay. The maternally inherited *EHMT2* variant causes multiple aberrant splicing events in a subset of transcripts (∼25%), including retention of 291 nucleotides from intron 18, which generates a nonsense variant in the canonical *EHMT2* transcript.

**Conclusion:**

Our findings support a role for *EHMT2* in an autosomal recessive neurodevelopmental disorder and allowed anticipatory guidance for the patient’s family.

## 1 Introduction

*EHMT2* encodes euchromatic histone-lysine N-methyltransferase 2, also known as G9a, a histone methyltransferase that facilitates the reversible mono- and dimethylation of histone H3 at lysine 9 (H3K9me1/2 methylation) (Tachibana et al., 2001). H3K9 methylation is a repressive mark involved in gene silencing and has been reported to influence DNA methylation, which is critical for proper regulation of gene expression and embryonic development (Tachibana et al., 2008; Guo et al., 2014; Messerschmidt et al., 2014).

EHMT2 functions as a heterodimer with the related histone methyltransferase euchromatic histone-lysine N-methyltransferase 1 (EHMT1, or GLP) (Tachibana et al., 2005). Both EHMT1 and EHMT2 contain a cysteine-rich region involved in protein-protein interactions, an ankyrin repeat domain, which acts as a “reader” domain to recognize and recruit the EHMT1/2 heterodimer to H3K9-methylated histones, and a SET domain, the catalytic “writer” domain, which is responsible for methyltransferase activity and for EHMT1/2 heterodimerization (Tachibana et al., 2005; Collins et al., 2008; Shinkai and Tachibana, 2011; Kerchner et al., 2021; Poulard et al., 2021). H3K9 methylation is primarily catalyzed by EHMT2, and EHMT1 facilitates recruitment of EHMT2 to chromatin in its role as a histone “reader” (Tachibana et al., 2008; Rots et al., 2024). Though EHMT1 and EHMT2 methylation activity is mainly focused on histones, they also have a variety of known non-histone substrates (Chen et al., 2010; Zhang et al., 2015; Rao et al., 2019). EHMT1 binds other proteins such as MPP8 to promote DNA methylation through recruitment of the DNA methyltransferase DNMT3A (Tachibana et al., 2008; Chang et al., 2011; Sanchez et al., 2021). EHMT2 can regulate CpG-rich promoters and methylates non-histone proteins, including CDYL, WIZ, ACIN1, DNMT1, HDAC1, ERCC6, KLF12, and TP53, as well as itself (Deimling et al., 2017; Auclair et al., 2016).

Deficiency of EHMT1 or EHMT2 causes global disruption of H3K9me1/2 methylation and is embryonically lethal in mice, suggesting that these genes are non-redundant and critical for proper embryonic development (Tachibana et al., 2002; Auclair et al., 2016). Heterozygous pathogenic variants in *EHMT1* cause Kleefstra syndrome type 1 (KS1, OMIM #610253), a recognizable autosomal dominant Mendelian syndrome characterized by global developmental delay, characteristic facial dysmorphisms, variable congenital anomalies, and behavioral abnormalities (Stewart et al., 2004; Kleefstra et al., 2005; 2006). Global methylation analysis of blood DNA from individuals with KS1 shows a distinguishable methylation episignature pattern, which can be used to clarify the molecular diagnosis in cases with no detectable *EHMT1* variants or variants of uncertain significance (VUS) (Aref-Eshghi et al., 2020).

Recently, three reports (Martinez-Delgado et al., 2024; Carvalho et al., 2025; Hnízda et al., 2025) described individuals harboring variants in *EHMT2* with phenotypes consistent with KS1. One individual with a homozygous splice site variant in *EHMT2* identified by exome sequencing presented with global developmental delay, dysmorphic features, congenital heart disease, and aggressive behavior (Carvalho et al., 2025). A second individual identified by exome sequencing as carrying a *de novo* heterozygous missense *EHMT2* variant in the catalytic SET domain exhibited similar, Kleefstra-like features and congenital heart disease (Martinez-Delgado et al., 2024). More recently, a larger case series reported six new individuals harboring *de novo* heterozygous *EHMT2* missense variants or in-frame deletions in the SET domain. These individuals presented with overlapping features of global developmental delay, craniofacial abnormalities, congenital heart disease, and structural brain abnormalities (Hnízda et al., 2025). All reported patients displayed a DNA methylation episignature that overlaps with that of KS1.

Though these reports support a role for *EHMT2* in a novel syndromic neurodevelopmental disorder, this gene is not yet recognized as disease-causing in the Online Mendelian Inheritance in Man (OMIM) database and the mode of inheritance (autosomal dominant *versus* recessive) is unclear, underscoring the importance of describing additional cases to support a role for *EHMT2* in human disease and establish mode of inheritance. Furthermore, the phenotypes and molecular findings of previously reported cases have not been systematically compiled and comparatively discussed.

Here, we present a male proband with global developmental delay and multiple congenital anomalies who was found by genome sequencing to harbor biallelic *EHMT2* variants, with at least one variant being likely hypomorphic, producing a reduced but not absent level of normal product compared to the wild-type allele. We provide molecular evidence from DNA methylation episignature analysis and RNA sequencing that supports a causal relationship between *EHMT2* variants and syndromic phenotypes. We further analyze the phenotypes and molecular findings in individuals with autosomal dominant *versus* recessive *EHMT2*-related neurodevelopmental disorder. This represents the second reported case of suspected *EHMT2*-related neurodevelopmental disorder with autosomal recessive inheritance and allowed for anticipatory guidance regarding recurrence risk.

## 2 Methodology

### 2.1 Trio genome sequencing

Proband underwent trio genome sequencing at Baylor Genetics Laboratory. Genome sequencing at Baylor Genetics includes genome-wide single nucleotide variant analysis, copy number variation analysis, structural variant analysis, mitochondrial DNA sequencing, select trinucleotide repeat expansion disorders, small and large insertions and deletions, and regions of homozygosity.

### 2.2 RNA sequencing

Total RNA was extracted from peripheral blood in an EDTA tube following standard procedures and processed using commercially available kits. cDNA was synthesized using Oligo (dT) and random primers. Targeted reflex RNA-sequencing was performed using an amplicon-based next-generation sequencing (NGS) approach using two different sets of primers covering all transcript junctions between exons 12–24 of *EHMT2* to avoid allele drop out and ensure result consistency (the variant of interest is at the 5′ end of intron 18). The amplicon products were used for library preparation using the KAPA HyperPrep kit, and then subject to sequencing using NovaSeq X. Samples were sequenced to an average depth of 150 million reads. Next-generation sequencing data was converted from BCL to FASTQ and processed using DRAGEN software. Mapping and aligning to the GRCh38 reference genome was performed using paired-end reads mapped with the DRAGEN RNA pipeline as described previously (Zhao et al., 2025). Aligned BAM files were visualized using the Integrative Genomics Viewer (IGV). Junction-spanning reads were required to meet a minimum coverage of >50×. Aberrant splicing was measured as the proportion of aberrant junction reads relative to constitutively spliced regions within the same sample, benchmarked against healthy controls (n = 2) processed under identical conditions. A threshold of ≥20% aberrant junction reads was applied to define a significant splicing event (internal data from Baylor Genomics Laboratory; Vaquero-Garcia et al., 2023).

### 2.3 DNA methylation episignature

DNA methylation data was generated using the clinically validated EpiSign v5 test available through Greenwood Genetics (Dong et al., 2008; Aref-Eshghi et al., 2019; Aref-Eshghi et al., 2020). Genomic DNA was extracted from blood of the proband and his parents, and a methylation array was performed to determine methylated and unmethylated signals. Data was generated using the v2 version of the MethylationEPIC array (Illumina Infinium MethylationEPIC BeadChip, v2.0, San Diego, CA, USA). Values were normalized and filtered using R (version 3.5.1). Beta values reflecting methylation levels were compared to the established EpiSign Knowledge Database, which consists of over 10,000 methylation profiles from healthy controls and established disease-specific cohorts - including KS1. A methylation variant pathogenicity (MVP) score was calculated for each sample, where 0 and one reflect discordant and concordant results, respectively. The proband’s methylation profile was compared algorithmically to the Episign Knowledge Database using the MVP scoring system to identify overlaps. Scores of greater than 0.5 were considered diagnostic (Sadikovic et al., 2021).

### 2.4 Comparative clinical and molecular analysis

Clinical and molecular data from the proband was compared in a structured manner with the only *EHMT2* cases reported in the literature to date, identified through PubMed-based literature searches (Martinez-Delgado et al., 2024; Carvalho et al., 2025; Hnízda et al., 2025; Table 1). Phenotypes were catalogued and reviewed by clinical (AS, TK) and laboratory (DR, LC, ACK, MJB) geneticists familiar with Kleefstra and Kleefstra-like syndromes. Phenotypic features were further assessed against those described in KS1, using frequency data from a previously published cohort of 172 individuals (Rots et al., 2024; Zdolšek Draksler et al., 2024). All variants were classified following the American College of Medical Genetics and Genomics guidelines and submitted to ClinVar. ClinVar accession numbers are displayed in Table 1.

**TABLE 1:** Molecular and clinical features of probands with *EHMT1* or *EHMT2* variants with evident phenotypic overlap.

​	Martinez-Delgado et al.	Hnizda et al.						Carvalho et al	Our proband	Autosomal dominant EHMT2-related disease summary	Autosomal recessive EHMT2-related disease summary	Kleefstra syndrome type I (OMIM #610253)
Number	1	2	3	4	5	6	7	8	9	​	​	​
Gene	*EHMT2*	*EHMT2*	*EHMT2*	*EHMT2*	*EHMT2*	*EHMT2*	*EHMT2*	*EHMT2*	*EHMT2*	*EHMT2*	*EHMT2*	*EHMT1*
Inheritance	Dominant	Dominant	Dominant	Dominant	Dominant	Dominant	Dominant	Recessive	Recessive	Dominant	Recessive	Dominant
Coding Sequence Change	c.3229G>T	c.3225_3236del	c.3229G>A	c.3335A>G	c.3472T>C	c.3487_3492del	c.3485A>G	c.328+2T>G	c.2648_2649del; c.2262_2265del	-----	-----	-----
Amino Acid Change	p.(Ala1077Ser)	p.(Glu1076Val1079del)	p.(Ala1077Thr)	p.(Asn1112Ser)	p.(Phe1158Leu)	p.(Ser1163_Lys1164del)	p.(Lys1162Arg)	p.(?)	p.(Glu883Glyfs*48); p.(?)	-----	-----	-----
ClinVar Accession	VCV002687731.3	VCV004057269.1	SCV007518740	VCV004057266.1	VCV004057267.1	VCV004057270.1	VCV004057268.1	VCV004813853.1	SCV007516937; SCV007495237	-----	-----	-----
Variant Type	Missense	Indel	Missense	Missense	Missense	Indel	Missense	Splice	Frameshift	Missense or in frame indel variants	Biallelic splice or frameshift variants	Various
Variant Location	SET Domain	SET Domain	SET Domain	SET Domain	SET Domain	SET Domain	SET Domain	N/A	Ankryrin repeat domain	SET Domain	-----	Various
Mechanism of Pathology	Suspected dominant negative	Suspected dominant negative	Suspected dominant negative	Suspected dominant negative	Suspected dominant negative	Suspected dominant negative	Suspected dominant negative	Suspected hypomorphic, loss-of-function	Suspected hypomorphic, loss-of-function	Suspected dominant negative	Suspected hypomorphic, loss-of-function	Loss-of-function
Functional Data	Episignature	Episignature	Episignature	Episignature	Episignature	Episignature	Episignature	Episignature and RNA-sequencing	Episignature and RNA-sequencing	Episignature	Episignature and RNA-sequencing	-----
Brachycephaly	+	+	+	-	+	-	+	+	+	5/7 (71%)	2/2 (100%)	Typical feature
Midface Flattening	+	+	+	+	+	-	+	+	+	6/7 (86%)	2/2 (100%)	Typical feature
Eyebrow Anomalies	+	-	-	+	-	+	+	+	+	4/7 (57%)	2/2 (100%)	Typical feature
Prognathism	-	+	+	+	+	-	+	+	+	5/7 (71%)	2/2 (100%)	Typical feature
Ptosis	+	-	-	-	-	-	-	+	+	1/7 (14%)	2/2 (100%)	-
Dental Anomalies	+	+	+	+	+	+	+	-	-	7/7 (100%)	0/2 (0%)	∼ 48%
Congenital Heart Disease	+	+	-	+	+	+	+	+	+	6/7 (86%)	2/2 (100%)	∼ 40%
Genitourinary Anomalies	+	+	+	+	-	+	-	-	+	5/7 (71%)	1/2 (50%)	∼ 5%
Umbilical Hernia	-	-	+	-	-	-	-	+	+	1/7 (14%)	2/2 (100%)	-
Spine Anomalies	+	-	-	-	-	-	-	+	+	1/7 (14%)	2/2 (100%)	∼ 5%
Hypotonia	+	+	+	-	+	+	+	+	+	6/7 (86%)	2/2 (100%)	∼ 38% at birth
Developmental Delay/Intellectual Disability	+	+	+	-	+	+	+	+	+	6/7 (86%)	2/2 (100%)	∼ 65%
Behavior Abnormalities	Stereotypy	Autistic Features	+	Hyperkinesis	-	-	Stereotypy	Agression	Autism-spectrum	-----	-----	∼ 67%

Variants are reported following the Human Genome Variation Society (HGVS) nomenclature using the transcript NM_006709.5.

### 2.5 Ethics statement

Consent for participation and publication was obtained from both parents. Both parents explicitly provided consent for publication of identifiable images. Mother of the proband participated in the international coordinating conference call. This study was approved by the Institutional Review Board at The Children’s Hospital of Philadelphia (IRB 16-013278) and was conducted in accordance with the Declaration of Helsinki.

### 2.6 CARE reporting guidelines

This case report was prepared in accordance with the CARE guidelines for clinical case reporting (Riley et al., 2017). The CARE checklist and a timeline summarizing key events are provided in Supplementary Figure S1; Supplementary Table S1.

## 3 Results

### 3.1 Clinical description and genetic findings

The patient is a 31-month-old male of Russian and Eastern European descent referred to Genetics for abnormal prenatal ultrasounds suggestive of small omphalocele, ventricular septal defect, short corpus callosum, inferior vermis hypoplasia, absent nasal bone, and absent gallbladder (Supplementary Figure S1). Mother was 30 years of age at the time of conception. She had a history of one prior pregnancy loss. To address the possibility of an underlying genetic syndrome, prenatal microarray (Integrated Genetics) and trio exome sequencing (GeneDx) were performed. A maternally inherited pathogenic variant in *CEP290*[NM_025114.3:c.4966 G>T; p.(Glu1656*)] was identified by exome sequencing. Given the zygosity and poor phenotypic overlap, this finding was considered consistent with carrier status for *CEP290*-related autosomal recessive disorders (Joubert syndrome, Leber congenital amaurosis, Meckel syndrome, Bardet-Biedl syndrome, and Senior-Loken syndrome) (Drivas et al., 2014).

He was born via vaginal delivery. Birth weight was 3.49 kg (47th percentile), length was 50.2 cm (53rd percentile), and head circumference was 34 cm (19th percentile). He was noted to have lower extremity cyanosis and an audible murmur. Echocardiogram demonstrated tricuspid valve dysplasia, ventricular septal defect, and a type B interrupted aortic arch. In addition to his congenital heart disease, he was found to have an umbilical hernia and not an omphalocele, a low-lying conus, right cryptorchidism, and chordee. Head ultrasound was suggestive of absence of the corpus callosum isthmus and splenium and inferior vermis hypoplasia. This was confirmed on subsequent brain MRI.

He returned for follow-up evaluation with the genetics service at 19 months of age. History was notable for hypotonia, recurrent otitis media requiring myringotomy tube placement, obstructive sleep apnea requiring nocturnal CPAP, recurrent respiratory infections, nephrocalcinosis, and scoliosis. He exhibited global developmental delay, with independent sitting achieved at 11 months. He was diagnosed with autism spectrum disorder at 18 months of age.

**FIGURE 1:**
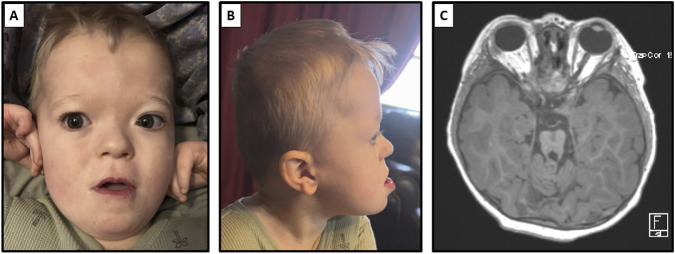
Clinical and neuroimaging features. **(A,B)** Frontal and profile views of the patient demonstrating microcephaly, brachycephaly, occipital flattening, midface flattening, hypertelorism, sparse medial eyebrows, and prognathism. **(C)** Brain MRI showing corpus callosum thinning with dysplastic fornices, and thickening of the irregular superior cerebellar peduncles with vermian dysplasia/hypoplasia and loss of the decussation of the superior cerebellar peduncles.

Growth parameters were notable for a weight of 14.9 kg (>99th percentile), height of 86.5 cm (80th percentile), and a head circumference of 44 cm (<1st percentile; corresponding to the 50th percentile for a 6-month-old). Physical examination was notable for plagiocephaly, square face, midface flattening, arched eyebrows, hypertelorism, a wide nasal root, open-mouth posturing with a protruding tongue, everted lower lip vermilion, prognathia, and hypotonia (Figures 1A,B).

**FIGURE 2:**
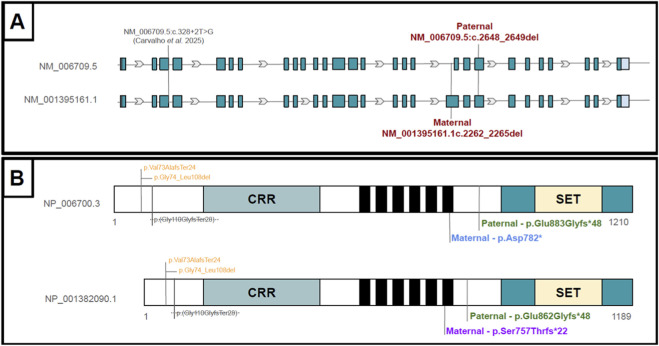
*EHMT2* variant mapping and predicted protein effects. **(A)** Schematic of the *EHMT2* gene (Upper: NM_006709.5; Lower: NM_001161.1). Proband’s compound heterozygous variants are indicated in red. The previously reported homozygous variant (Carvalho et al., 2025) is shown in black. The heterozygous *de novo* variants associated with autosomal dominant disease reported by Hnízda et al. (2025) localize to the SET domain. **(B)** Schematic of the EHMT2 protein (Upper: NP_006700.3; Lower: NP_001382090.1). EHMT2 consists of a cysteine-rich region (CRR - amino acids 420–549), an ankyrin repeat domain (black bars, amino acids 649–879), pre- and post-SET domains (turquoise, amino acids 972–1035 and 1161–1080, respectively), and a catalytic SET domain (yellow, amino acids 913–1193). The RNA-sequencing–based predicted (gray with a dashed line through it) *versus* identified (orange) effects of the previously-reported *EHMT2* splice variant are shown (Carvalho et al., 2025). For our proband, the predicted effect of the paternally inherited variant is shown in green. The maternally inherited variant affecting only the very low-expression isoform (NP_001382090.1) is shown in purple. The RNA-sequencing–based predicted effect of the maternal variant on the canonical transcript (NM_006709.5) is shown in blue, introducing a premature stop codon.

Re-analysis of exome sequencing data (GeneDx) was recommended and was notable only for the previously detected maternally inherited *CEP290* variant. To address a possible Joubert syndrome diagnosis, brain MRI was performed to assess for molar tooth sign, the pathognomonic MRI finding in Joubert syndrome (Drivas and Bennett, 2014). Brain MRI showed thickening of the superior cerebellar peduncles with vermian hypodysplasia and loss of the superior cerebellar peduncle decussation (Figure 1C), findings interpreted as not consistent with Joubert syndrome. Trio genome sequencing was performed (Baylor Genetics Laboratory) and demonstrated biallelic variants of uncertain significance in *EHMT2* [NM_006709.5:c.2648_2649del p.(Glu883Glyfs*48), inherited from the father; NM_006709.5:c.2344-19_2344-16del r.spl, inherited from the mother] (Figures 2A,B).

The patient is currently 31 months old. Independent walking was achieved at 28 months. He follows one-step commands, speaks a few single words, and has multiple signs and gestures for communication. He receives speech, occupational, and physical therapy, applied behavior analysis therapy, and he receives special needs instruction. The clinical and molecular data of the patient described in this manuscript are summarized in Table 1, alongside previously reported patients harboring *EHMT2* variants, and compared to the frequency of the same clinical features in a KS1 cohort.

### 3.2 RNA-sequencing identifies aberrant transcripts from the maternal EHMT2 allele

In addition to the canonical transcript (NM_006709.5), *EHMT2* has multiple alternate transcripts that generate different protein isoforms. The paternal variant NM_006709.5:c.2648_2649del is predicted to cause a frameshift in all *EHMT2* isoforms. The maternally-inherited variant causes a 4-bp deletion in intron 18 of the canonical transcript NM_006709.5 that might affect splicing and overlaps with a coding region causing a frameshift only in the reported NM_00139516.1 isoform (NP_001382090.1: Ser757Thrfs*22), which is reported to be expressed at very low levels in most human tissues (Figure 2; Supplementary Figure S2). This variant is predicted by SpliceAI to create a cryptic acceptor site (acceptor gain Δ score = 0.29), suggesting a potential impact on normal splicing with incomplete penetrance. This *in silico* prediction prompted us to investigate *EHMT2* transcripts by RNA-sequencing.

**FIGURE 3:**
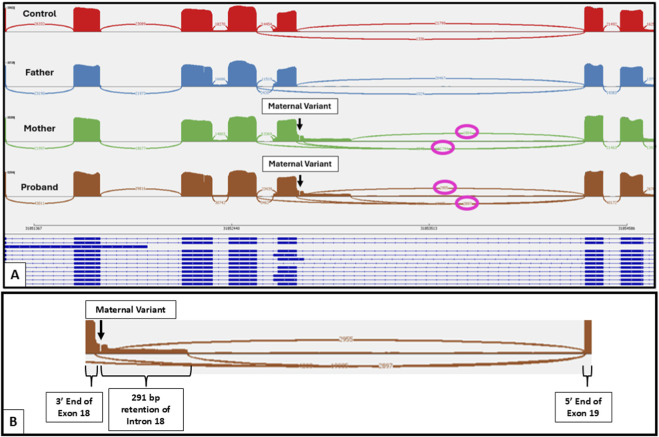
RNA-Seq analysis of *EHMT2* splicing. **(A)** Sashimi plots of RNA-sequencing data generated from blood of a control individual, the proband, and the proband’s parents. The black arrow indicates the novel splice variant produced by the maternal *EHMT2* variant allele, which results in inclusion of 291 nucleotides of intron 18, introducing a premature stop codon. Pink circles indicate abnormal splicing isoforms. **(B)** Magnification of the exon-intron boundaries between exons 18 and 19 of the maternal allele demonstrating the alternate splice allele that results in retention of 291 nucleotides of intron 18.

RNA-sequencing data analysis demonstrated that the maternally inherited NM_006709.5:c.2344-19_2344-16del variant results in multiple aberrant splice isoforms (25% of reads; Figures 3A,B), including an event resulting in retention of 291 nucleotides of intron 18, leading to a nonsense variant in the canonical *EHMT2* transcript [NM_006709.5:r.2343_2344ins291; p.(Asp782*)], which likely triggers nonsense mediated decay (Figures 2A,B). The paternally inherited variant was not associated with alternate splicing events. Though the amplicon-based assay used in this study is not designed for absolute quantification of allele-specific transcript abundance, analysis did reveal reduced abundance of the variant-bearing transcript derived from the paternal allele (Supplementary Figure S3). The paternal aberrant transcript introduces a frameshift allele predicted to generate a premature termination codon, which is a well-established trigger for nonsense mediated decay, though reduced RNA production or stability via a different mechanism cannot be formally excluded.

### 3.3 Genome-wide DNA methylation episignature analysis demonstrates overlap with KS1

**FIGURE 4:**
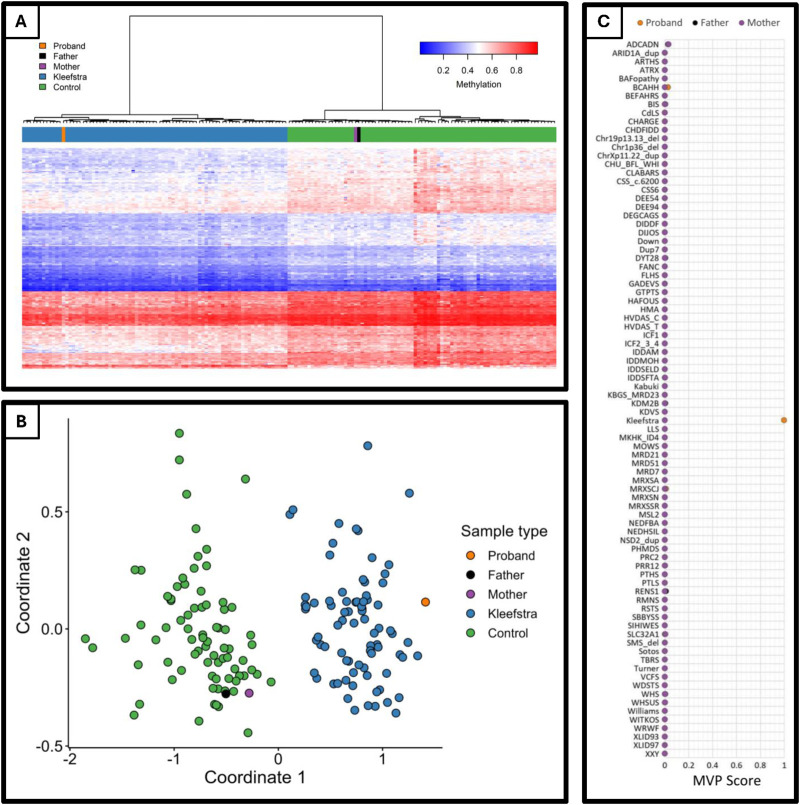
Genome-wide DNA methylation analysis shows a DNA methylation profile that overlaps with KS1 in the proband but not in parents. **(A)** Hierarchical clustering and **(B)** Multidimensional scaling plots showing that the proband (orange) has a DNA methylation profile that clusters with individuals carrying the confirmed Kleefstra profile (blue) that is distinct from controls (green). The parental samples (black and purple) cluster with the control group, indicating profiles similar to unaffected cases. **(C)** Graph depicting MVP scores, a multi-class supervised classification system capable of discerning multiple DNA methylation episignature patterns by generating a probability score for each signature. The elevated Kleefstra score in the proband (1) indicates a DNA methylation profile consistent with Kleefstra cases, while the zero scores in the parental samples indicate profiles more similar to controls.

Methylation episignature profiles of the parents clustered with healthy controls, whereas the proband clustered with KS1 patients (Figures 4A,B). The MVP scores indicated a high overlap between the proband’s DNA methylation episignature profile and the KS1 profile and very low overlap with other rare syndromes in the EpiSign DNA Methylation Knowledge Database (Figure 4C). As expected, the parents scored low for all syndromes.

## 4 Discussion

Here we report a proband carrying biallelic *EHMT2* variants who presented with facial features suggestive of KS1, global developmental delay, and multiple congenital anomalies. DNA methylation episignature profiling demonstrated a strong overlap with the KS1 signature, which is in line with previous reports of individuals with *EHMT2* variants having DNA methylation episignatures that overlap with that of KS1 (Martinez-Delgado et al., 2024; Carvalho et al., 2025; Hnízda et al., 2025).

Although the role of *EHMT2* in Mendelian neurodevelopmental disease remains incompletely established, prior studies support its importance in embryonic development, DNA methylation, and transcriptional regulation (Tachibana et al., 2002; Auclair et al., 2016; Poulard et al., 2021). The clinical case described in this manuscript represents the second report involving biallelic variants in *EHMT2*, supporting a role for *EHMT2* in a neurodevelopmental syndrome with an autosomal recessive mode of inheritance.

Probands with both autosomal dominant and autosomal recessive *EHMT2*-related neurodevelopmental disorder share common phenotypes including neurodevelopmental delay, dysmorphic features, and musculoskeletal, heart, and kidney defects, similar to KS1, making them largely phenotypically indistinguishable. It is possible that genitourinary, dental, and cardiac abnormalities are more common in *EHMT2*-related neurodevelopmental disorders, present in 6, 7, and 8 of 9 reported probands, respectively (67%, 78%, 89%) compared to the respective 5%, 48%, and 40% prevalence reported in KS1. This conclusion is tentative based on limited sample size and requires validating cohort expansion studies (Table 1; Rots et al., 2024). These shared phenotypes combined with the overlapping episignatures in individuals with KS1 or autosomal dominant/recessive *EHMT2*-related neurodevelopmental disorders support a common mechanism of disease pathogenesis. Further work is required to determine whether the episignatures of KS1 and autosomal dominant and recessive *EHMT2*-related neurodevelopmental disorder can be distinguished.

Notably, previously reported cases with suspected autosomal dominant inheritance involve heterozygous missense and in-frame deletion variants affecting the SET domain that presumably compromise EHMT2 enzymatic activity via a dominant-negative effect, whereas variants associated with recessive modes of inheritance do not appear to follow this domain-specific distribution and involve truncating and splice variants that likely represent hypomorphic alleles, i.e., alleles with partial loss of function (Carvalho et al., 2025).

These observations suggest that distinct molecular mechanisms may underlie the dominant and recessive *EHMT2*-related presentations. We hypothesize that *EHMT2* missense variants do not interfere with protein production and dimerization; however, the incorporated catalytically dead EHMT2 protein may disrupt the function of the entire protein complex, causing disease *via* a dominant-negative mechanism, as described in a recent study (Hnízda et al., 2025). In contrast, we hypothesize that the splicing/frameshift variants producing either unstable or truncated EHMT2 protein observed in recessive cases may reduce the amount of function EHMT2 while preserving sufficient residual function to remain compatible with life.

Consistent with this model, in the previously-reported autosomal recessive *EHMT2* case, a cryptic splice site was identified that could rescue the reported *EHMT2* splice variant, and RNA-sequencing studies in blood confirmed the use of two cryptic splice sites, one of which maintains the protein reading frame (Carvalho et al., 2025). For our proband, RNA-sequencing data suggests that the maternally inherited variant does not completely abolish normal splicing of the canonical *EHMT2* transcript, consistent with a hypomorphic allele, though we cannot eliminate the possibility that these splicing patterns are unique to the evaluated tissue (blood), not reflecting the biology in clinically relevant tissues such as the brain, nor can we exclude the possibility that additional genetic modifiers, including variants in related regulatory genes, may influence penetrance, expressivity, or residual EHMT2 pathway activity. Though supporting immunoblot data from patient-derived samples demonstrating reduced EHMT2 protein levels and functional studies showing sequelae of dysregulated H3K9 methylation in our proband would be helpful in supporting a loss-of-function mechanism for autosomal recessive *EHMT2*-related neurodevelopmental disorder, ethical considerations must take precedence and preclude further basic science studies. Despite limitations, this model would explain the strong phenotypic overlap between *EHMT1* and autosomal dominant/recessive *EHMT2*-related neurodevelopmental disorders, with all pathology caused by impaired activity of the EHMT1/2 heterodimer due to *EHMT1* haploinsufficiency (KS1), partial *EHMT2* deficiency (autosomal recessive *EHMT2*-related disease), or via dominant negative interference (autosomal dominant *EHMT2*-related disease).

In summary, we provide further evidence supporting *EHMT2* as a causal gene for a Kleefstra-like neurodevelopmental syndrome with multiple congenital anomalies and an autosomal recessive inheritance pattern. Our case supports a role for *EHMT2* in human disease and provides additional data to clarify both the autosomal dominant and recessive inheritance patterns described for this gene. Most importantly, our studies provided a molecular answer to a family seeking anticipatory guidance and recurrence risk counseling for family planning.

## Data Availability

The *EHMT2* variants reported in this study have been submitted to ClinVar under accession numbers SCV007495237 and SCV007516937. Data beyond those included in this manuscript are not publicly available due to patient privacy restrictions but may be provided by the corresponding authors upon reasonable request and subject to institutional approval.
