# Supplementary figures and images for: A role for EHMT2 in a novel autosomal recessive neurodevelopmental syndrome? A case report
Source: Front Genet. 2026 Jun 26;17:1824138. doi: 10.3389/fgene.2026.1824138 (PMC13350482; doi:10.3389/fgene.2026.1824138)

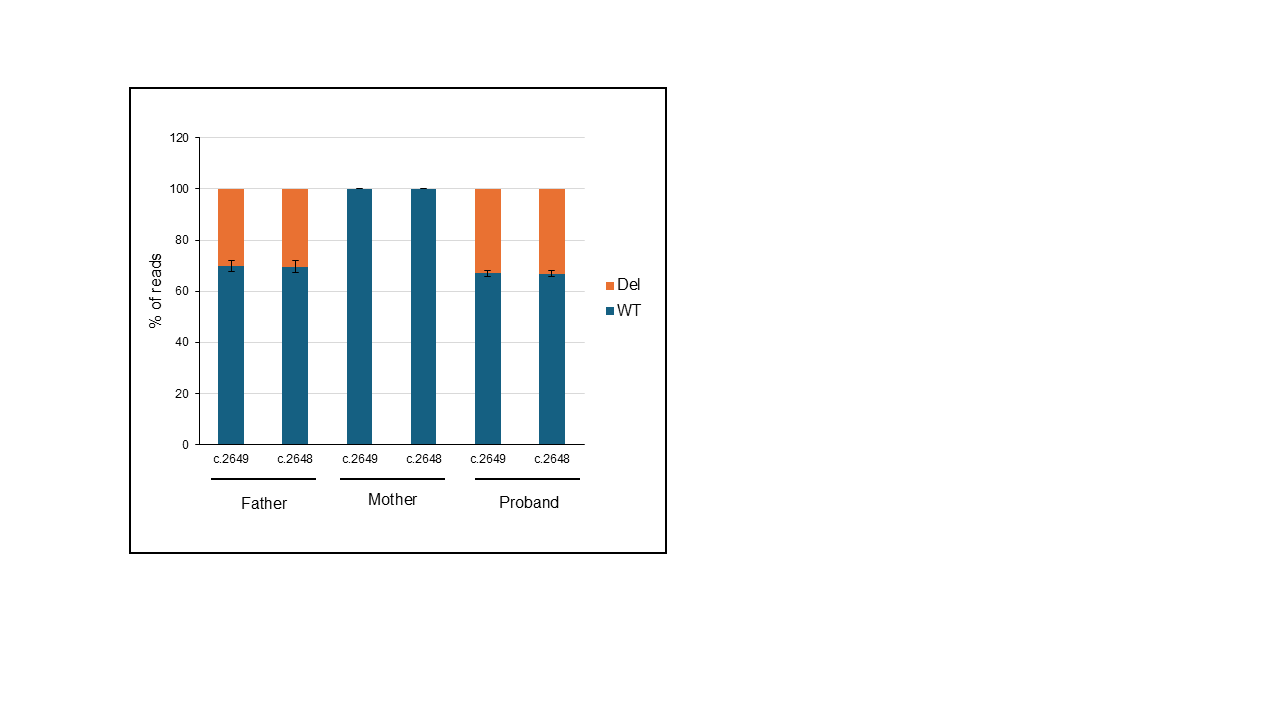

Supplement: Supplementary file 1 [file Image3.tif]

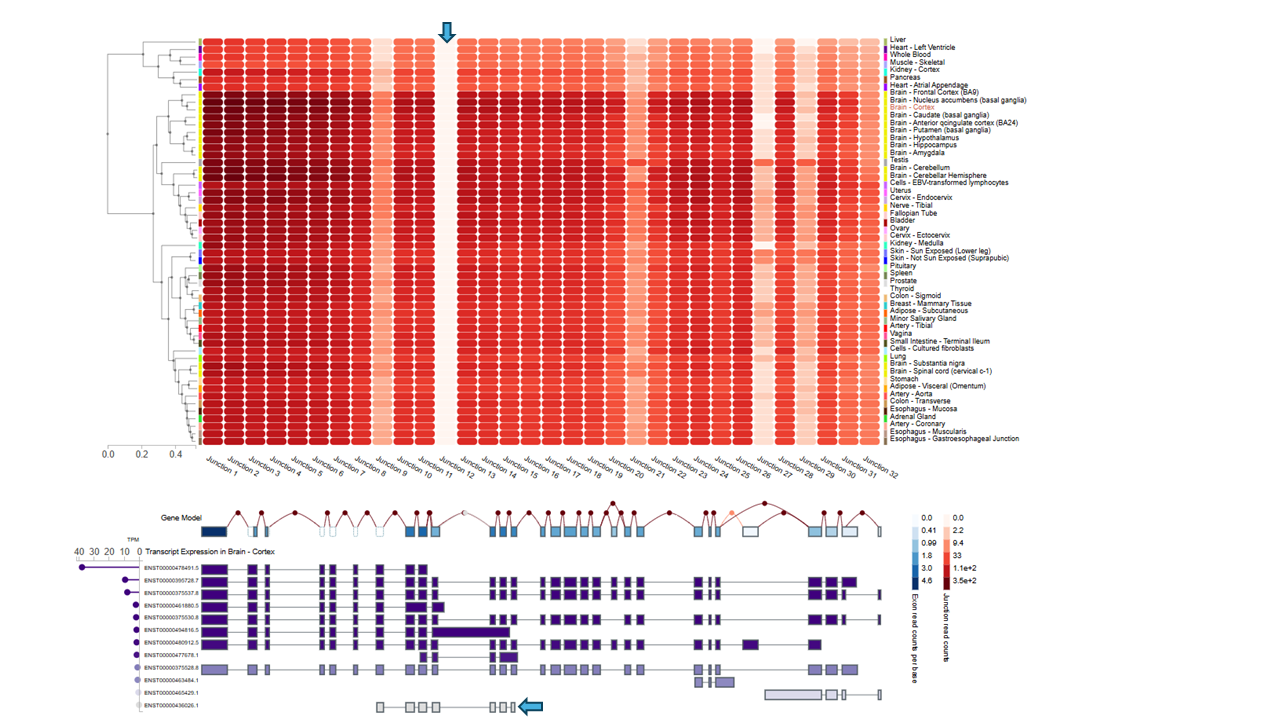

Supplement: Supplementary file 2 [file Image2.tif]

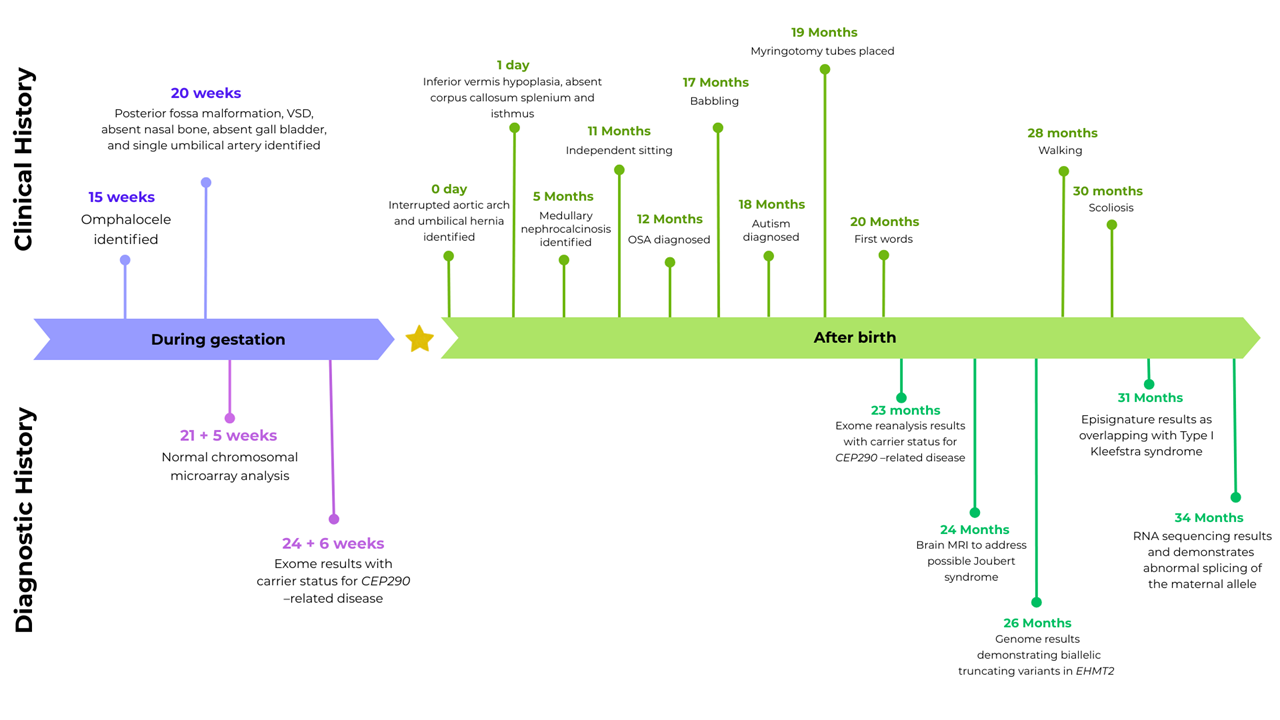

Supplement: Supplementary file 3 [file Image1.tif]
